# Defining the fetal origin of MLL-AF4 infant leukemia highlights specific fatty acid requirements

**DOI:** 10.1016/j.celrep.2021.109900

**Published:** 2021-10-26

**Authors:** Vasiliki Symeonidou, Hélène Jakobczyk, Salem Bashanfer, Camille Malouf, Foteini Fotopoulou, Rishi S. Kotecha, Richard A. Anderson, Andrew J. Finch, Katrin Ottersbach

**Affiliations:** 1 Centre for Regenerative Medicine, University of Edinburgh, Edinburgh, EH16 4UU, UK; 2 Leukaemia Translational Research Laboratory, Telethon Kids Cancer Centre, Telethon Kids Institute, University of Western Australia, Perth, WA 6009, Australia; 3 MRC Centre for Reproductive Health, The Queen’s Medical Research Institute, University of Edinburgh, Edinburgh EH16 4TJ, UK; 4 Cancer Research UK Edinburgh Centre, Institute of Genetics and Molecular Medicine, University of Edinburgh, Edinburgh EH4 2XU, UK

**Keywords:** MLL-AF4, infant leukemia, acute lymphoblastic leukemia, B-ALL, ELOVL1, PLK1, onco-fetal signature

## Abstract

Infant MLL-AF4-driven acute lymphoblastic leukemia (ALL) is a devastating disease with dismal prognosis. A lack of understanding of the unique biology of this disease, particularly its prenatal origin, has hindered improvement of survival. We perform multiple RNA sequencing experiments on fetal, neonatal, and adult hematopoietic stem and progenitor cells from human and mouse. This allows definition of a conserved fetal transcriptional signature characterized by a prominent proliferative and oncogenic nature that persists in infant ALL blasts. From this signature, we identify a number of genes in functional validation studies that are critical for survival of MLL-AF4+ ALL cells. Of particular interest are PLK1 because of the readily available inhibitor and ELOVL1, which highlights altered fatty acid metabolism as a feature of infant ALL. We identify which aspects of the disease are residues of its fetal origin and potential disease vulnerabilities.

## Highlights

• Fetal blood progenitors display a more proliferative and oncogenic nature
• This onco-fetal signature is maintained in infant leukemia blasts
• Fetal progenitors and blasts share specific fatty acid requirements
• Fetal programs maintained in blasts may be exploited for therapies

## Graphical abstract

**Figure:**
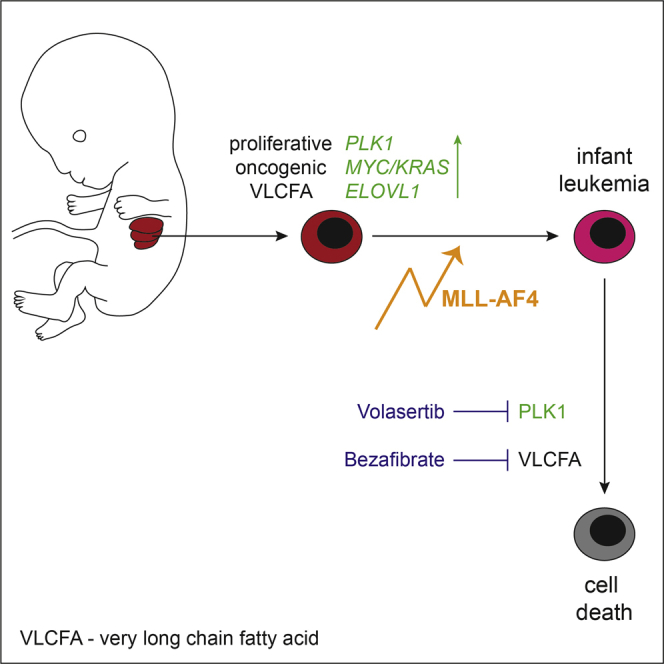

## Teaser

Infant leukemias have a unique biology and originate in fetal blood progenitors. Symeonidou et al. show that fetal blood progenitors display a proliferative and oncogenic signature that persists in infant leukemia blasts and is required for disease maintenance. Among these fetal-derived disease contributors is a requirement for specific fatty acids.

## Introduction

Infant MLL-AF4-driven pro-B acute lymphoblastic leukemia (ALL) is the most common leukemia in infants (Bonaventure et al., 2017; Meyer et al., 2018). It arises *in utero* and is an aggressive disease with a dismal prognosis (Ford et al., 1993; Meyer et al., 2018; Pieters et al., 2019; Sanjuan-Pla et al., 2015). The latest Interfant study (Interfant-06) demonstrated that the prognosis of infants has not improved, and there is an urgent need for innovative therapeutic strategies (Pieters et al., 2019).

Infant MLL-AF4+ ALL is initiated by a chromosomal translocation, fusing chromosome 4 to 11 (t(4;11)) and generating the highly potent MLL-AF4 oncofusion. In two recent studies, it has been shown that the t(4;11) translocation is the only molecular abnormality identified in the majority of patients (Agraz-Doblas et al., 2019; Andersson et al., 2015). This clean mutational landscape suggests that the complexity of this disease is attributable to the fusion protein and the fetal origin of the leukemia-initiating cell. Despite this genetic simplicity, it has proven particularly difficult to develop faithful disease models (Ottersbach et al., 2018), indicating that, for identification of specific therapeutic targets, a better understanding of the biology of the disease is required.

It has become apparent that there are fundamental differences between fetal and adult cells with regard to their proliferative behavior, lineage bias, and the way in which they respond to disease-associated mutations (Benz et al., 2012; Bowie et al., 2007; Mascarenhas et al., 2016). Importantly, fetal cells are more prone to transformation by Mll-AF4 compared with their adult counterparts (Barrett et al., 2016). The fetal origin of the leukemia-initiating cell thus plays a critical role in disease pathogenesis. Two recent studies have investigated human fetal hematopoiesis in detail, shedding light on the processes taking place during these early developmental stages (O’Byrne et al., 2019; Popescu et al., 2019). Although these studies are valuable resources, they have not led to identification of novel therapeutic targets for infant MLL-AF4 ALL.

In this study, we focus on the fetal origin of the disease and describe a novel approach that allowed us to identify new disease targets for infant MLL-AF4+ ALL. By comparing the transcriptome of fetal cells, neonatal/adult cells, and blasts (in humans and mice), we identified a molecular signature that was conserved between fetal tissue and blasts. The common genes were targeted pharmacologically and genetically, which showed that a large number were critical for disease maintenance, validating our approach. We identified *PLK1* and *ELOVL1* as key genes for survival of MLL-AF4+ cells.

## Results

### The fetal transcriptome is characterized by a proliferative and oncogenic nature

**Figure 1:**
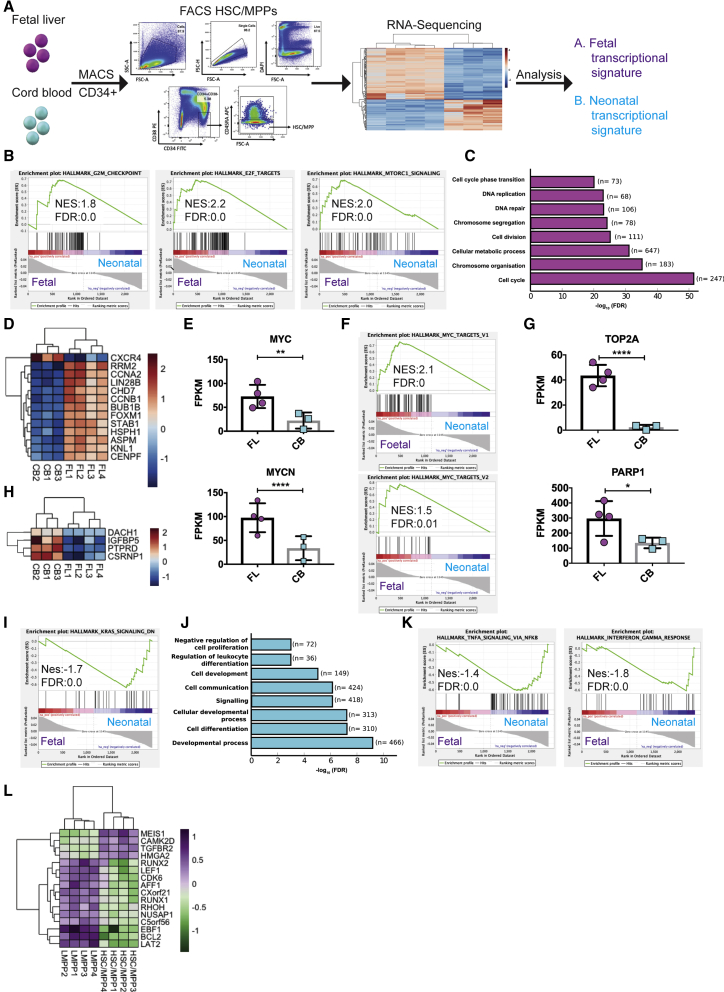
FL-derived HSC/MPPs are characterized by an over-proliferative and oncogenic nature (A) Experimental design. HSC/MPPs were sorted as CD34+CD38−CD45RA−. (B) GSEA of genes overrepresented in FL HSC/MPPs. (C) GO of processes overrepresented in FL HSC/MPPs. n = number of genes overrepresented for a specific process. (D) Heatmap of genes linked previously with cancer or leukemia identified in the top 25 DE genes. (E) Expression of MYC and MYCN in FL and CB HSC/MPPs. RNA-seq data are shown as mean ± SD; each dot represents a sample. (F) GSEA of genes overrepresented in FL HSC/MPPs. (G) Expression of TOP2A and PARP1 in FL and CB HSC/MPPs. RNA-seq data are shown as mean ± SD; each dot represents a sample. (H) Heatmap of tumor suppressor genes overrepresented in the CB samples. (I) GSEA of genes overrepresented in CB HSC/MPPs. (J) GO of processes overrepresented in CB HSC/MPPs. (K) GSEA of genes overrepresented in CB HSC/MPPs. (L) Heatmap of DE genes between HSC/MPPs and LMPPs, which are in common with SEM cell MLL-AF4-spreading targets. FDR, false discovery rate; FPKM, fragments per kilobase of transcript per million mapped reads; NES, normalized enrichment score. ^∗^p < 0.05, ^∗∗^p < 0.01, ^∗∗∗^p < 0.001, ^∗∗∗∗^p < 0.0001. See also Figure S1 and Table S1.

Infant MLL-AF4+ ALL arises *in utero*; therefore, we speculated that studying differences between fetal and neonatal tissues would allow us to understand how the unique origin of this disease affects its phenotype. We performed RNA sequencing (RNA-seq), comparing human second trimester fetal liver (FL) with developmentally more mature cord blood (CB) hematopoietic stem cells (HSCs) and progenitor cells (multipotent progenitors [MPPs]) (HSC/MPPs; CD34+CD38−CD45RA−) (Figure 1A). We chose this immature cell population because the fact that leukemic blasts often co-express lymphoid and myeloid markers and are able to undergo lineage switching suggests that the disease initiates in an uncommitted progenitor. We identified 2,394 differentially expressed (DE) genes between the two populations, with 1,162 more highly expressed in FL cells (Table S1, tab 1).

Gene set enrichment analysis (GSEA) and Gene Ontology (GO) revealed the proliferative nature of the fetal cells (Figures 1B and 1C), as exemplified by an upregulation of G2M checkpoint and E2F targets and a number of cyclins and cyclin-dependent kinases (Figure S1A); genes associated with chromosome segregation during mitosis, such as members of the Aurora kinase complex (Figures S1B and S1C); and condensins and cohesins (Figure S1D). There was also an enrichment in the MTORC1 signaling pathway, a master growth regulator (Figure 1B; Dowling et al., 2010). To functionally validate the more proliferative nature of fetal cells, human FL and CB CD34+ cells were plated in methylcellulose under myeloid conditions. There was no significant difference between the total numbers of colonies (Figure S1E), suggesting a similar enrichment for hematopoietic stem and progenitor cells (HSPCs) in the CD34+ population, although there were fewer of the more mature BFU-E in the FL CD34+ population and a trend toward higher numbers of the most immature CFU-GEMM type. Notably, there was a significantly higher number of cells per colony (Figure S1F) and per plate (Figure S1G), confirming FL HSPCs as being more proliferative.

In addition, many genes and pathways more highly expressed in FL HSC/MPPs have been linked previously to cancer or leukemia, including 12 of the top 25 DE genes (Figure 1D). Importantly, this included the proto-oncogenes *MYC* and *MYCN* (Figure 1E), and GSEA revealed an enrichment of MYC targets (Figure 1F). Among the genes overrepresented in FL cells were *TOP2A* and *PARP1* (Figure 1G). These genes are of particular interest for MLL-AF4+ ALL as they encode enzymes that play a central role in chromosomal translocations (Cowell and Austin, 2012; Wray et al., 2013).

Contrary to FL cells, CB HSC/MPPs showed higher expression of a number of tumor suppressor genes and lower KRAS signaling (Figures 1H and 1I). The KRAS proto-oncogene and its associated pathways are frequently mutated in cancer (Nigro et al., 1989; Tsuchida et al., 2016). The general transcriptional profile of neonatal cells revealed upregulation of processes related to the immune system, including tumor necrosis factor alpha (TNF-α) signaling via nuclear factor κB (NF-κB) and interferon gamma response, which could reflect the more mature nature of these cells or an inflammatory response (Figures 1J and 1K).

These data suggest that the fetal origin of the leukemia-initiating cell with its proliferative and oncogenic nature could be a fundamental contributor to the aggressive nature of the infant disease.

### FL-derived LMPPs express key MLL-AF4 target genes

In a recent study, Agraz-Doblas et al. (2019) showed that blasts from infant patients share a similar transcriptional profile with HSPCs (Lin−CD34+CD38−) which include HSC/MPPs and lymphoid-primed MPPs (LMPPs); however, they were unable to observe a closer transcriptional match between blasts and HSC/MPPs or LMPPs. To further look into this question, we investigated the transcriptional differences between FL HSC/MPPs and LMPPs (Table S1, tab 2). As expected, LMPPs upregulated a number of genes required for lymphoid commitment, including AF4 (*AFF1*), whereas HSC/MPPs had higher *MLLT3* (AF9), another common MLL fusion partner (Figure S1H). A closer look at the genes more highly expressed in HSC/MPPs revealed stem cell signature genes such as *MEIS1*, *HOXB2*, and *HMGA2* (Figure S1I). Although the two populations had a multitude of differences, we observed that none of the DE genes was reflective of a more proliferative nature of one population over the other, a key finding when considering consequences of each one of them being the cell of origin of the disease.

We next compared the DE genes between HSC/MPPs and LMPPs with the spreading targets identified by Kerry et al. (2017). They describe a unique MLL-AF4 binding pattern spreading into the gene body of disease-relevant target genes and leading to increased transcription. We observed a larger number of genes in common between the spreading targets and LMPPs, including well known MLL-AF4 targets such *BCL2*, *RUNX1*, *RUNX2*, and *CDK6* and a much lower number for HSC/MPPs (Figure 1L, Godfrey et al., 2017; Krivtsov et al., 2008; van der Linden et al., 2014; Wilkinson et al., 2013). It is interesting that a number of key MLL-AF4 targets were expressed at a higher level in LMPPs. Whether this is relevant to the question of the cell of origin or disease phenotype deserves further investigation.

### Identification of conserved disease-relevant genes

**Figure 2:**
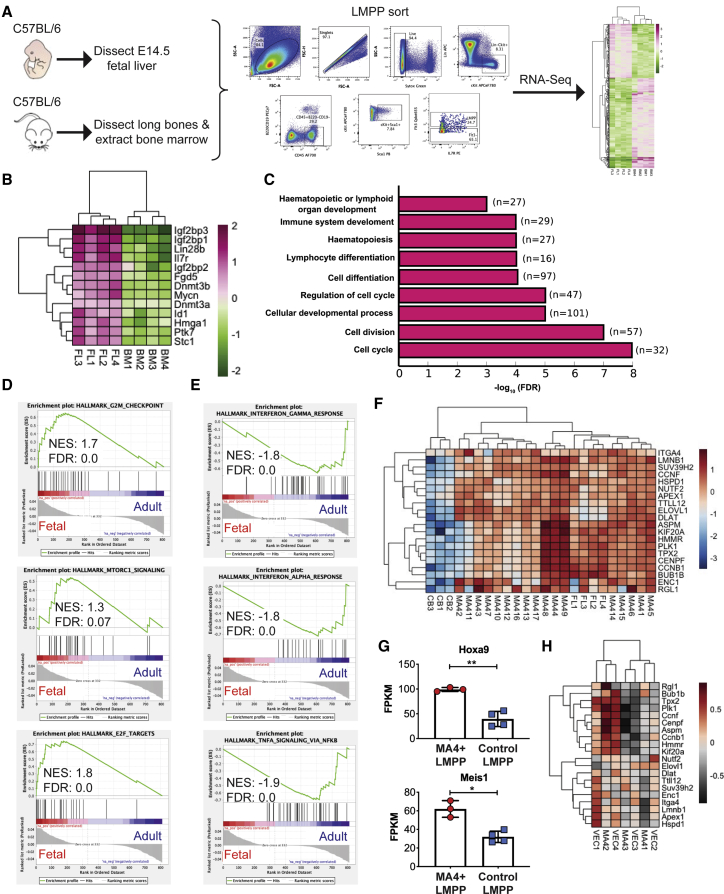
The onco-fetal signature is conserved across humans and mice (A) Experimental design. LMPPs were sorted as Lin−Sca1+ckit+CD45+B220−CD19−Flt3+. (B) Heatmap of oncogenes overrepresented in FL LMPPs. (C) GO of genes more highly expressed in FL LMPPs. (D) GSEA of genes overrepresented in FL LMPPs. (E) GSEA of genes more highly expressed in BM LMPPs. (F) Heatmap of genes conserved across species and maintained in MLL-AF4+ infant ALL blasts. (G) *Hoxa9* and *Meis1* expression in murine FL Mll-AF4+ LMPPs and control LMPPs. RNA-seq data are shown as mean ± SD; each dot represents a sample. ^∗^p < 0.05, ^∗∗^p < 0.01. (H) Heatmap of the 20 genes selected for further analysis as represented in murine FL LMPPs expressing Mll-AF4 (MA4) and control LMPPs (VEC). See also Figure S2 and Table S1.

Important regulators are likely to be conserved across species. Therefore, we performed RNA-seq comparing murine FL LMPPs with adult bone marrow (BM) LMPPs (Figure 2A; Table S1, tab 3). As with human fetal cells, we observed an upregulation of the oncogene *Mycn* and other genes linked to cancer and leukemia (Figure 2B). In fact, a number of these are well-known onco-fetal genes, such as *Lin28b*, the Igf2bp family, and the Hmga family of proteins (He et al., 2018; Helsmoortel et al., 2016; Marquis et al., 2018; Palanichamy et al., 2016; Roy et al., 2013; Stoskus et al., 2016). In particular, HMGA2, LIN28B, and IGF2BP1 have been referred to as an “oncogenic triangle” that is critical for cancer initiation (Busch et al., 2016) and that was also co-ordinately overrepresented in our data (Figure 2B). Furthermore, Lin28b and Hmga2 have been reported to be more highly expressed in fetal HSCs and to be important for their enhanced self-renewal compared with adult HSCs (Copley et al., 2013). Similar to human fetal cells, cell cycle/proliferation-associated genes were notably more enriched in mouse fetal cells (Figure 2C), with GSEA also highlighting G2M checkpoint, E2F targets, and MTORC1 signaling upregulation in mouse FL LMPPS (Figure 2D), whereas adult LMPPs were enriched in immune function genes (Figure 2E). To identify DE genes conserved across species, we defined the intersection of the DE genes of our murine and human datasets (i.e., genes common between Table S1, tabs 1 and 3). We identified 62 genes commonly overrepresented in the FL-derived populations of mouse and human and 55 common in the neonatal/adult datasets (Table S1, tab 4; Figure S2A).

To extract genes that might be critical for disease maintenance, we investigated expression of the 62 common FL genes in blasts from individuals with MLL-AF4+ ALL (Andersson et al., 2015; Table S4, tab 1). We observed that 20 genes were expressed at similar levels between fetal cells and blasts, whereas the remaining appeared to be downregulated (Figure 2F; Figure S2B). To ensure that the genes commonly overrepresented in fetal cells and blasts were due to the fetal origin and not a consequence of MLL-AF4 expression, we performed an RNA-seq experiment comparing murine E14 FL LMPPs expressing Mll-AF4 with control LMPPs. At this early developmental stage, cells have not been fully transformed, and the transcriptional and phenotypic changes induced by Mll-AF4 are representative of early Mll-AF4 targets in a pre-leukemic stage (Barrett et al., 2016). Differential expression analysis revealed upregulation of *Hoxa9* and *Meis1* (Figure 2G), genes that are well-known targets of MLL-AF4; however, none of the 20 selected genes were upregulated in the Mll-AF4-expressing LMPPs (Figure 2H). These data suggest that the 20 genes, commonly overrepresented in fetal cells and blasts, are a residue of the fetal origin of the disease and not a consequence of the MLL-AF4 fusion.

### *PLK1* is highly expressed in the blasts of infant patients and required for cell survival

**Figure 3:**
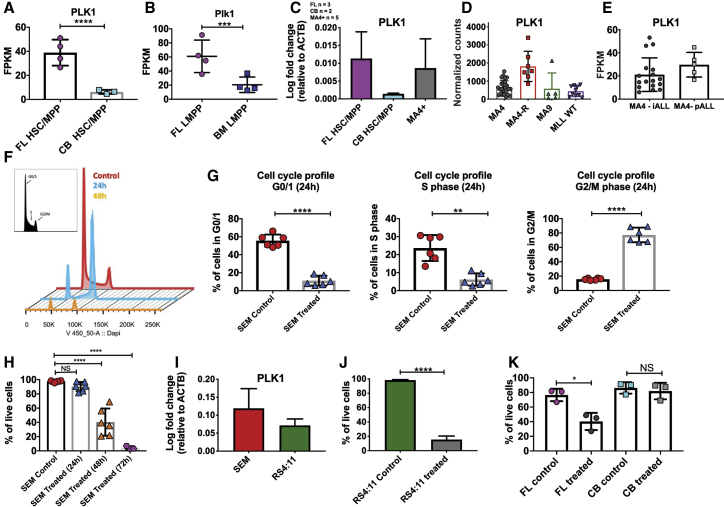
PLK1 regulates proliferation and survival in MLL-AF4+ leukemic cells (A and B) *PLK1* expression in human FL and CB HSC/MPPs (A) and murine FL and BM LMPPs (B). RNA-seq data are shown as mean ± SD; each dot represents a sample. (C) qPCR of *PLK1* expression in human FL and CB HSC/MPPs and infant MLL-AF4+ ALL blasts (MA4+). FL, n = 3; CB, n = 2; MA4+, n = 5. Data are shown as mean + SD. (D and E) *PLK1* expression in the RNA-seq datasets by (D) Agraz-Doblas et al. (2019) and (E) Andersson et al. (2015). MA4, MLL-AF4; MA4-R, relapsed; MA9, MLL-AF9; MLL wild type (WT), MLL germline B-ALL; iALL, infant B-ALL; pALL, pediatric B-ALL. (F) Cell cycle profile of SEM cells. Red, SEM cells vehicle-treated control; blue, SEM cells treated with 50 nM volasertib for 24 h; orange, SEM cells treated with 50 nM volasertib for 48 h. (G) Cell cycle profile of cells treated with 50 nM of volasertib after 24 h. Data are shown as mean ± SD. Student’s t test was performed. (H) Viability of cells treated with 50 nM of volasertib. Data are shown as mean ± SD. ANOVA was performed. (I) qPCR of *PLK1* expression in SEM and RS4;11 cells; n = 3. Data are shown as mean + SD. Student’s t test was performed. (J) Viability of RS4;11 cells treated with 50 nM volasertib for 72 h. Data are shown as mean +SD. Student’s t test was performed. (K) CD34+ FL and CB cells treated with 50 nM volasertib for 48 h (FL, n = 3; CB, n = 3). Data are shown as mean ± SD. Student’s t test was performed. NS, not significant. ^∗^p < 0.05, ^∗∗^p < 0.01, ^∗∗∗^p < 0.001, ^∗∗∗∗^p < 0.0001. See also Table S4.

Among the genes commonly overrepresented in FL cells and infant blasts was *PLK1*, a member of the serine/threonine protein kinases that is critical for cell cycle progression (Figures 3A–3C). PLK1 has been implicated in a number of acute leukemias (Bhojwani et al., 2006; Chow et al., 2017; Renner et al., 2009) and is expressed in a range of infant and pediatric ALLs, with levels being particularly high in samples from relapsed infants with MLL-AF4+ ALL (Figures 3D and 3E). Given its importance in cancer/leukemia, a number of PLK1 inhibitors have been developed and clinically tested for their safety (Gjertsen and Schöffski, 2015; Gopalakrishnan et al., 2018; Kobayashi et al., 2015; Rudolph et al., 2009). To investigate whether an inhibitor could be used to treat infant MLL-AF4+ ALL, we exposed SEM cells (a cell line derived from a pediatric individual with MLL-AF4 ALL) to 50 nM of the PLK1 inhibitor volasertib, a concentration that did not have cytotoxic effects in healthy natural killer (NK) cells (Gopalakrishnan et al., 2018). 24 h after treatment, there was an arrest in the G2/M phase of the cell cycle, resulting in decreased numbers of cells in the G0/1 and S phases (Figures 3F and 3G). 48 h after treatment, half of the SEM cells were apoptotic, and at 72 h, very few viable cells remained (Figure 3H). This effect was not unique to SEM cells because we observed similar results in RS4;11, an adult MLL-AF4+ ALL cell line that expressed similar levels of *PLK1* (Figures 3I and 3J). This prompted us to investigate the effect of *PLK1* inhibition on primary tissues, where we observed that CB CD34+ cells are unaffected by volasertib (Figure 3K). Strikingly, their fetal counterparts are sensitive to the same treatment, which not only confirms the enhanced vulnerability of fetal cells to the inhibitor because of higher PLK1 expression but also suggests that normal HSPCs of infant patients, who are developmentally more CB like, would be unaffected by volasertib treatment—an encouraging observation that deserves further investigation.

### ELOVL1 is a novel disease target for infant MLL-AF4-driven ALL

To identify additional fetal-specific genes that continue to be important for disease maintenance, we incorporated a CRISPR-Cas9 screen, for which we established a SEM-Cas9-GFP-expressing cell line and performed knockout studies of 19 genes, that were overrepresented in the FL population (human and mice) and were expressed at similar levels in the blasts (Tables S1, Tabs 1, 3, and 4; Figure 2F). This genetic screen included genes with a variety of different functions, such as cell cycle/proliferation (*ASPM*, *TPX2*, *APEX1*, *CCNB1*, *CCNF*, *KIF20A*, *CENPF*, and *BUB1B)*, cell migration (*HMMR*, *ITGA4*, *ENC1*, and *LMNB1*), chromatin binding (*SUV39H2*), pyruvate metabolism (*DLAT*), fatty acid metabolism (*ELOVL1*), protein transport into the nucleus (*NUTF2*), molecular chaperones (*HSPD1)*, and two less well-characterized genes (*RGL1* and *TTLL12)*.

**Figure 4:**
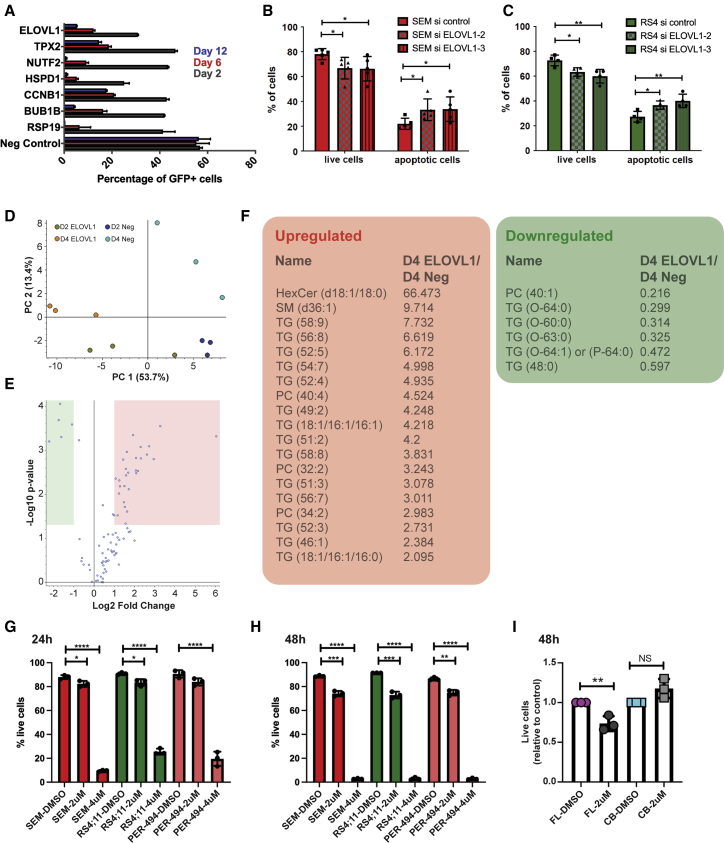
ELOVL1-regulated lipid metabolism is required for MLL-AF4+ leukemic cell survival (A) CRISPR-Cas9 competition assay. Data are shown as mean + SD; n = 2. (B and C) Viability of SEM (B) and RS4;11 cells (C) after knockdown of *ELOVL1* expression with two different siRNA constructs. “Live” cells are AnnexinV− Sytox−, and “apoptotic” cells are AnnexinV+ Sytox+/−. Data are shown as mean ± SD. 2-way ANOVA was performed. (D) Principal Component Analysis (PCA) of the lipidomics experiment results. ELOVL1, ELOVL1 knockout; Neg, knockout with negative sgRNA. (E) Volcano plot of D4 ELOVL1 knockout compared with D4 neg knockout. Lipids are shown as dots; red, upregulated; green, downregulated. (F) Lipids highlighted in red were upregulated and are predominantly polyunsaturated lipids. Lipids highlighted in green were downregulated and are lipids with saturated and monosaturated fatty acid chains. TG, triglyceride; SM, sphingomyelin; HexCer, hexosyl ceramides; PC, phosphatidylcholines. The first numeric value indicates the number of carbons and the second the number of double bonds. (G and H) Viability of SEM (pediatric), RS4;11 (adult), and PER-494 (infant) MLL-AF4+ B-ALL cell lines treated with DMSO (control, diluent) and bezafibrate (BF) at the indicated concentrations after 24 h (G) and 48 h (H). Data are shown as mean ± SD. ANOVA was performed. (I) Viability of primary human FL and CB cells treated for 48 h with 2 μM BF. Data are shown as mean ± SD. Student’s t test was performed. ^∗^p < 0.05, ^∗∗^p < 0.01, ^∗∗∗^p < 0.001, ^∗∗∗∗^p < 0.0001. See also Figures S3 and S4 and Tables S2 and S3.

To investigate the effects of the knockout of these genes, we performed a competition assay where SEM-Cas9-expressing cells were mixed with wild-type cells before addition of the sgRNAs. With this approach, we are able to identify genes important for survival of SEM cells because knockout would decrease the percentage of Cas9-GFP+ cells, whereas wild-type cells remain unaffected (Figures S3A and S3B). We discovered 6 genes (*ELOVL1*, *TPX2*, *NUTF2*, *HSPD1*, *CCNB1*, and *BUB1B*) whose knockout resulted in apoptosis of SEM-Cas9 cells (Figure 4A). To select genes unique to the infant disease, we filtered out genes that had been identified in previously published whole-genome CRISPR screens of other cancers and leukemias (Erb et al., 2017; Hart et al., 2015; Wang et al., 2017): *TPX2*, *NUTF2*, *CCNB1*, and *BUB1B*.

Two genes were unique to the infant disease: *HSPD1* and *ELOVL1*. We investigated their expression in healthy BM blood cells using the Human Cell Atlas (Hay et al., 2018) (http://www.altanalyze.org/ICGS/HCA/splash.php), which showed that *HSPD1* was highly expressed in all blood cell types and, therefore, not an ideal gene to target (Figure S3C). In contrast, *ELOVL1* showed low expression in BM cells, making it an attractive candidate target for the infant disease. This more restricted expression pattern of *ELOVL1* was confirmed in the BloodSpot dataset (https://servers.binf.ku.dk/bloodspot/), showing expression in a subset of mature myeloid cells, whereas *HSPD1* was detected at all levels of the hematopoietic hierarchy (Figures S3D and S3E). To ensure that the ELOVL1 knockout effect was not due to off-target effects, we confirmed sgRNA efficiency in a TIDE assay (Figure S3F), which determines the type and frequency of targeted mutations generated by CRISPR/Cas9 at the *ELOVL1* locus.

*ELOVL1* was overrepresented in FL cells (human and murine) and expressed at similar levels in human FL HSC/MPPs and infant blasts (Figures S3G–S3I). It was also expressed in a range of infant and pediatric B-ALL subtypes (Figures S3J and S3K) and in three MLL-AF4+ cell lines: SEM, RS4;11, and PER-494 cells (Cheung et al., 2021), derived from a pediatric, adult, and infant MLL-AF4+ individual, respectively (Figure S3L). To confirm the requirement for ELOVL1 in survival of leukemic cells (Figure 4A), we performed knockdown studies with two different small interfering RNA (siRNA) constructs in SEM and RS4;11 cells (Figure S4A). In both cell lines, reducing ELOVL1 levels resulted in decreased viability (Figures 4B and 4C). Interestingly, preliminary data suggest that overexpression of *ELOVL1* has no effect on SEM cell survival (Figures S4B and S4C).

ELOVL1 is an enzyme involved in lipid metabolism; specifically, elongation of very-long-chain fatty acids (VLCFAs). To investigate the mechanism of action of ELOVL1 in ALL and obtain a global picture of which lipids were affected by the knockout, a lipidomics experiment was performed (Table S2). SEM-Cas9 cells transduced with *ELOVL1*-specific sgRNA and a control (negative) sgRNA were collected on days 2 and 4, and their lipidomes were compared. Principal Component Analysis revealed a clear separation of the different populations (Figure 4D). There were no differentially represented lipids between day 2 SEM-Cas9 cells transduced with sgRNA for *ELOVL1* and the control. On day 4, however, a small number of lipids was downregulated (Figure 4E, highlighted in green) and a larger number of lipids was upregulated (Figure 4E, highlighted in red). Further analysis revealed that the most significantly differentially represented lipids were downregulated and contained very long saturated and monounsaturated fatty acid chains, whereas overrepresented lipids were unsaturated with multiple double bonds (Figure 4F; Table S2). This was in accordance with previous publications that show ELOVL1 to be critical for elongation of saturated VLCFAs, specifically of the type containing up to 26 carbons (C26:0), and monosaturated VLCFAs, including sphingolipids, which are critical structural components of cellular membranes (Ohno et al., 2010; Tvrdik et al., 2000). It has been shown that bezafibrate (BF), proven to be safe for use in humans, reduces C26:0 levels without affecting *ELOVL1* expression levels (Engelen et al., 2012; Miller and Spence, 1998). We therefore used BF to test whether synthesis of VLCFAs is important for leukemogenesis. We initially conducted dose-response experiments (Figure S4D) in the three MLL-AF4+ leukemia cell lines to determine IC_50_ values and subsequently used BF in viability assays at 2 μM and 4 μM, which is 100- to 200-fold lower than what has been used previously in fibroblast cultures (Engelen et al., 2012). After 24 h treatment with 2 μM BF, there was a reduction in viability in all three cell lines, with a further reduction after 48 h, where most cells had died at the higher concentration of 4 μM (Figures 4G and 4H). Importantly, and akin to the PLK1 inhibition results, 48 h treatment of primary human FL cells resulted in a similar reduction in cell viability, whereas primary human CB cells were unaffected (Figure 4I).

These findings suggest that there are different requirements for VLCFAs and plasma membrane maintenance between fetal and neonatal/adult HSPCs that are maintained in the blasts of infants with MLL-AF4 ALL.

## Discussion

Our transcriptomic data indicate that, in humans and mice, fetal cells are characterized by a proliferative and oncogenic nature, an extremely permissive environment for initiation of the aggressive infant disease. In contrast, neonatal and adult cells showed higher expression of a number of tumor suppressor genes and immune system processes. It was interesting to find KRAS signaling downregulated in neonatal cells because its role in MLL-AF4+ leukemia has often been debated. Although sequencing studies have confirmed a silent mutational landscape for infant MLL-AF4+ ALL, RAS-activating mutations are detected frequently and have been associated with a poorer prognosis and with extramedullary leukemia in mouse models; however, the presence of RAS mutations does not appear to be required, and they are often subclonal and absent at relapse (Agraz-Doblas et al., 2019; Andersson et al., 2015; Prieto et al., 2016). One could speculate that activating RAS mutations aid leukemogenesis from a cellular origin where RAS signaling is normally downregulated (e.g., from the neonatal stage), whereas this would not be required in cases where the translocation occurs in cells that have naturally high KRAS activity.

There has been much speculation about the potential cell of origin for MLL-AF4+ infant ALL. Although data from the mouse (Barrett et al., 2016; Malouf and Ottersbach, 2018) pointed to LMPPs, and we found a higher number of MLL-AF4-spreading genes expressed in LMPPs, the signatures identified in the HSC/MPP population in our study make this an equally vulnerable population to transformation. The disease may initiate from either but might then result in slight phenotypic differences. Indeed, we have recently described a subclassification of MLL-AF4+ infants, with evidence pointing toward these possibly having a different developmental origin (Symeonidou and Ottersbach, 2021).

Our final 20 conserved candidate genes were expressed at similarly high levels in MLL-AF4+ infant blasts, which was not a consequence of MLL-AF4 expression. Seven of them (*LMNB1*, *HSPD1*, *APEX1*, *DLAT*, *HMMR*, *CENPF*, and *ITGA4*), however, have been shown to be direct MLL-AF4 targets by chromatin immunoprecipitation sequencing (ChIP-seq), which may help to maintain their expression during transformation (Kerry et al., 2017). Of particular interest among those was PLK1 because there is a clinically safe approved inhibitor (volasertib) available, and high expression of this gene in infant blasts suggests that it could be a promising therapeutic target. It has been tested in dose escalation trials with pharmacokinetic studies for acute myeloid leukemia (AML), chronic myeloid leukemia, and myelodysplastic syndrome and in children (2–18 years of age) with various cancers, but, to our knowledge, is not currently being investigated for infant ALL and, therefore, represents a promising new avenue. The fact that normal CB cells are insensitive to the inhibitor at the same dose is encouraging.

The majority of the genes selected have been shown to be critical for survival of multiple cancers and leukemias, and their potential as therapeutic targets in infant ALL should be investigated further. However, we decided to focus our attention on genes that appear to be unique to infant MLL-AF4-driven ALL and with low expression in healthy BM hematopoietic cells, with the most prominent candidate being *ELOVL1*. Another indication for a potential role of this gene in infant leukemia came from a study by Wang et al. (2017), in which they performed a whole-genome CRISPR screen of different AML cell lines. One of the cell lines was THP1, which is derived from an infant patient. From their data, it was clear that ELOVL1 knockout had a greater effect on survival of the infant cell line compared with the ones derived from adult patients. Although direct evidence is still lacking, it is believed that ELOVL1 knockout exerts its apoptotic effect through a reduction in sphingolipids, which, in turn, alters the cell membrane environment, leading to activation of apoptotic pathways (Kihara, 2012). Interestingly, an increase in saturated VLCFAs, as would be achieved through upregulation of ELOVL1, has been linked to multidrug resistance (Kopecka et al., 2020). This is particularly interesting because infant patients have a very poor response to chemotherapy with a high incidence of relapse (Pieters et al., 2019). It may therefore not be surprising if the cell membrane of infant blasts had a different composition that renders it more resistant to standard chemotherapy. Our BF experiments certainly suggest that leukemia cells and fetal cells are more dependent on VLCFA synthesis. Future experiments will investigate whether the lipid composition of infant blasts differs from that of adult cells. Changes in lipid metabolism have become a well-recognized phenomenon in different types of cancers (Snaebjornsson et al., 2020), opening up new treatment strategies; however, this has not yet been explored in the context of infant ALL, making it an exciting new field of investigation.

By determining which aspects of the disease are residues of its fetal origin, we identified novel potential disease vulnerabilities, suggesting that the fetal origin of the disease could be its Achilles’ heel.

### Limitations of study

Although our study has identified a number of promising candidate facilitators of infant leukemia pathogenesis, the type of comparisons we made may have resulted in important regulators to be missed. As mentioned, there is substantial evidence pointing to the LMPP as a likely cell of origin, and we would have liked to have compared human fetal LMPPs with CB LMPPs. Unfortunately, because of technical issues with frozen starting material, very low LMPP numbers from CB samples, and poor-quality RNA, we have been unable to include CB LMPPs. Additional fetus-specific genes might also have emerged if we had been able to compare the transcriptome of human fetal HSPCs with that of human adult BM HSPCs, as was done for the mouse comparison. It is, however, very difficult to obtain normal human BM for scientific studies. There is also evidence that the cell of origin for infant leukemia may vary, resulting in different disease phenotypes, and that it includes cell populations outside of the HSPC compartment, such as a still unidentified, developmentally earlier mesodermal progenitor (Menendez et al., 2009; Symeonidou and Ottersbach, 2021) or a human fetal BM-specific early lymphoid progenitor (O’Byrne et al., 2019). It would be important to include these in future profiling studies. Although integration of mouse and human sequencing data highlighted conserved and therefore likely relevant fetal regulators, this comparison could equally have caused filtering out of important human-specific disease contributors because it has become apparent that the mouse cellular context is less conducive to lymphoblastic leukemia transformation (Lin et al., 2016; Malouf et al., 2021).

Our functional validation studies have largely relied on *in vitro* studies with human leukemia cell lines. These allow easy manipulation and consistency but may introduce artifacts as a result of their immortalization, high proliferative status, and the fact that they have often been perpetuated in culture over many years. Further investigation of our candidate regulators must therefore involve primary human patient samples and *in vivo* assays. This would then establish the potential of these regulators for further pre-clinical studies.

## STAR★Methods

### Key resources table

**Table:** 

REAGENT or RESOURCE	SOURCE	IDENTIFIER
**Antibodies**		

Human CD34-FITC (clone 581)	BioLegend	Cat#343503
Human CD38-PE (clone HIT2)	BioLegend	Cat#980302
Human CD45RA-APC (clone HI100)	BioLegend	Cat#304107
Mouse Sca1-PB (clone E13-161.7)	BioLegend	Cat#122519
Mouse ckit- APCeF780 (clone 2B8)	eBioscience/ThermoFisher Scientific	Cat#47-1171-80
Mouse CD45-AF700 (clone 30-F11)	eBioscience/ ThermoFisher Scientific	Cat#56-0451-82
Mouse Flt3-biotin (clone A2F10)	eBioscience/ ThermoFisher Scientific	Cat#13-1351-81
Mouse B220-PECy7 (clone RA3-6B2)	BioLegend	Cat#103221
Mouse CD19-PECy7 (clone 6D5)	BioLegend	Cat#115520
Mouse CD3e-APC (clone I45-2C11)	BioLegend	Cat#100312
Mouse Ter119-APC (clone TER119)	BioLegend	Cat#116212
Mouse F4/80-APC (clone 2BM8)	BioLegend	Cat#123116
Mouse Nk1.1-APC (clone PK136)	BioLegend	Cat#108709
Mouse Gr1-APC (clone RB6-8C5)	BioLegend	Cat#108412

**Biological samples**		

Human CD34+ cord blood cells	Scottish Cord Bank, Glasgow	N/A
Human CD34+ cord blood cells	Cambridge Stem Cell Biobank	N/A
Human CD34+ cord blood cells	Stem Cell Technologies	Cat#70008.3
Human fetal livers	Royal Infirmary of Edinburgh	N/A
Subcloning efficiency competent cells – DH5a	Life Sciences	Cat#18265017

**Chemicals, peptides, and recombinant proteins**		

StAV-Qd655	ThermoFisher Scientific	Cat#Q10123MP
SytoxGreen	ThermoFisher Scientific	Cat#S7020
Volasertib-BI6727	Cambridge Bioscience	Cat#b3300
Bezafibrate	Sigma-Aldrich	Cat#72516
PE-Annexin V	BioLegend	Cat#640908

**Critical commercial assays**		

iScript Advanced cDNA Synthesis Kit	BioRAD	Cat#1725038
PrimeScript 1st strand cDNA Synthesis Kit	Takara	Cat#6110A
DNeasy Blood & Tissue Kit	QIAGEN	Cat# 69506
NucleoSpin RNA kit	Macherey-Nagel	Cat# 740955
SMARTer® Stranded Total RNA-Seq Kit v1	Takara	Cat# 634411
SMARTer® Stranded Total RNA-Seq Kit - Pico Input Mammalian	Takara	Cat#635005
PLK1 TaqMan assay	Life Technologies	Hs00983227_m1
ELOVL1 TaqMan assay	Life Technologies	Hs00967955_g1
BACT TaqMan assay	Life Technologies	Hs01060665_g1

**Deposited data**		

Raw and normalized RNA-Seq data	This paper	GEO: GSE167234

**Experimental models: Cell lines**		

SEM (human pediatric MLL-AF4+ B-ALL)	Prof. Brian Huntly	DSMZ ACC 546
RS4;11 (human adult MLL-AF4+ B-ALL)	Prof. Brian Huntly	ATCC®CRL-1873
PER-494 (human infant MLL-AF4+ B-ALL)	Dr Rishi Kotecha	Cheung et al., 2021
HEK293T	In-house	ATCC®CRL-3216

**Experimental models: Organisms/strains**		

Mouse wild-type C57BL/6NCrl	Charles River UK	N/A
Mouse inducible Mll-AF4 invertor on C57BL/6NCrl	Prof. Terry Rabbitts	Metzler et al., 2006
Mouse VEC-Cre on C57BL/6NCrl	Prof. Nancy Speck	Chen et al., 2009

**Oligonucleotides**		

ELOVL1 sg1 forw: TCTCCTTTCCAGAGAGGTTCAG	IDT	N/A
ELOVL1 sg1 rev: GTGCTTTTTCCACCAAAGGTAG	IDT	N/A
gRNAs see Table S8	IDT	N/A
ELOVL1-targeted siRNA-1	ThermoFisher Scientific	s34992
ELOVL1-targeted siRNA-2	ThermoFisher Scientific	s34993
Negative control siRNA	Sigma-Aldrich	SIC001
VECP-F-219 (genotyping VEC-Cre mice) CCCAGGCTGACCAAGCTGAG	IDT	N/A
Cre-R107 (genotyping VEC-Cre mice) GCCTGGCGATCCCTGAACATG	IDT	N/A
MLL non-inverted (genotyping Mll-AF4 mice) TCGCCTTCTTGACGAGTTCT	IDT	N/A
MLL intron 10 (genotyping Mll-AF4 mice) ATGATGCCACTGTGCTGTGT	IDT	N/A

**Recombinant DNA**		

Cas9-GFP	Chen et al., 2015	Addgene: 63592
sgRNA-BFP	Yusa Kosuke	N/A
MD2-G	Addgene	Addgene: 12259
psPAX2	Addgene	Addgene: 12260

**Software and algorithms**		

Compound Discoverer for lipidomics	Thermo Scientific	N/A
GraphPad Prism	GraphPad software corporation	N/A
Trimmomatic	Bolger et al., 2014	N/A
Kallisto (v0.43.1)	Bray et al., 2016	N/A
Tximport (v.3.5)	Soneson et al., 2015	N/A
DESeq2 pipeline	Love et al., 2014	N/A
GSEA Jana Desktop tool	Subramanian et al., 2005	N/A
Gene Ontology analysis with Panther	Mi et al., 2019	N/A

### Resource availability

#### Lead contact

Further information and requests for resources and reagents should be directed to and will be fulfilled by the lead contact, Katrin Ottersbach (katrin.ottersbach@ed.ac.uk).

#### Materials availability

This study did not generate new unique reagents.

### Experimental model and subject details

#### Human sample preparation

Anonymized fetal tissue was obtained from morphologically normal 13-19 week fetuses following elective medical termination of pregnancy at the Royal Infirmary of Edinburgh after informed written consent (approved by the Lothian Research Ethics Committee, Reference: 08/S1101/1). Dissected fetal livers (FLs) were mechanically disrupted to create single cell suspensions. Mononucleated cells (MNC) were isolated from FL using Ficoll (Sigma-Aldrich) and further enriched with MACS CD34+ ultra-pure kit (Miltenyi Biotec). CD34-enriched cord blood (CB) samples were obtained from the Scottish Cord Bank in Glasgow, the Cambridge Stem Cell Biobank and Stem Cell Technologies.

cDNA from infant MLL-AF4+ patient samples for qPCR was kindly provided by Dr Pablo Menendez and Dr Ignacio Varela. Clinical data for these human patient samples are provided in Table S4, tab 2. All patients were enrolled in the Interfant-99 trial.

#### Murine sample preparation

All mouse work was carried out under a UK Home Office-approved license and following local ethical review. Animal husbandry followed institutional guidelines.

Mouse embryos (both male and female) were obtained from timed matings between C57BL/6NCrl mice, with the day of plug detection considered as embryonic day 0.5. E14.5 FLs were dissociated by drawing through a needle. Adult bone marrow (from 8-10 week old male and female mice) was collected by crushing the femora and tibia.

Conditional Mll-AF4 invertor mice (Metzler et al., 2006) were crossed with VEC-Cre transgenic mice (Chen et al., 2009) in order to induce Mll-AF4 expression in all definitive hematopoietic cells throughout ontogeny. Mll-AF4 expressing embryos had a Mll-AF4+ VEC-Cre+ genotype whereas the control embryos for these experiments were Mll-AF4- VEC-Cre+.

Genotyping of Mll-AF4 invertor and VEC-Cre transgenic mice was performed with the HotSHOT method (Truett et al., 2000) where an ear notch or a small portion of the embryo head was placed in 0.04% disodium EDTA and 0.25 NaOH in water and incubated at 95°C for 20 minutes at 1000rpm, followed by the addition of a neutralization reagent containing 4% 1M Tris-HCl in water. 1 μL of the mixture was mixed with 12.5 μL Kapa2G PCR mixture (Merck) and 9 μL of water and primers (sequences are available in Key Resources Table). The mixture was placed in a thermal cycler with the following program: 1) 92°C for 2min, 2) 95°C for 15sec, 3) 58°C for 15sec, 4) 72°C for 5sec (steps 2-4 were repeated for 30 cycles and 35 cycles for Mll-AF4 and VEC-Cre, respectively), 5) 72°C for 10sec. The PCR products were visualized on a 1% agarose (Sigma-Aldrich) using GelRed (Biotium).

#### Cell lines

The SEM cell line is of human B cell precursor leukemia and was originally derived from a 5-year-old girl at relapse of B-ALL with a t(4;11) translocation. The RS4;11 cell line is of human B cell precursor leukemia and was originally derived from a 32-year-old woman at relapse of B-ALL with a t(4;11) translocation. The PER-494 cell line was derived from an 11-month old female infant at diagnosis with MLL-AF4+ B-ALL (Cheung et al., 2021). SEM and RS4;11 cell lines were cultured in RPMI (ThermoFisher Scientific), 10% Fetal Calf Serum (FCS), 1% Penicillin/Streptomycin (Pen/Strep). 293T cells were cultured in GMEM (Life Technologies), 10% FCS, 1% Pen/Strep, 2mM L-glutamine, 0.1 mM MEM Non-Essential Amino Acids (Life Technologies). PER-494 were grown in RPMI (Thermofisher Scientific), 20% FCS, 2mM L-Glutamine, 1X non-essential amino acids, 1mM sodium pyruvate and 50uM 2-Mercaptoethanol.

### Method details

#### Fluorescence-activated cell sorting

Human cells were sorted on a FACS Aria III Fusion (BD) with CD34-FITC (clone 581), CD38-PE (clone HIT2) and CD45RA-APC (clone HI100), all from BioLegend, and DAPI (Sigma-Aldrich) for dead-cell exclusion. Hematopoietic stem and progenitor cells (HSC/MPPs) were sorted as CD34+CD38-CD45RA- and LMPPs as CD34+CD38-CD45RA+. 2,000 – 10,000 cells were sorted per sample.

Murine FL and BM LMPPs were sorted as Lin-Sca1+c-kit+CD45+B220-CD19-Flt3+ as described previously (Barrett et al., 2016), using a BD FACS ARIA II or BD FACS Fusion cell sorter. Lineage cocktail contained the following antibodies CD3e, Ter119, F4/80, Nk1.1, Gr1. 900-7,000 cells were sorted per sample. Antibody details are provided in the Key resources table.

#### RNA extraction, library preparation and sequencing

RNA extraction was performed immediately after sorting using a NucleoSpin® RNA kit (Macherey-Nagel). Libraries for RNA paired-end sequencing were prepared using SMARTer® Stranded Total RNA-Seq Kit v1 and 2-Pico Input Mammalian (Takara). 1ng of high-quality RNA (RIN ≥ 8) and 15 PCR cycles were used for the amplification of the library.

Human data were obtained with Illumina NextSeq 100bp paired-end RNA-sequencing (HiSeq 4000) at Beijing Genomics Institute, while murine data were obtained with Illumina NextSeq 75bp paired-end RNA-seq (HiSeq 4000) at Edinburgh Genomics.

#### RNA sequencing analysis pipelines

Raw reads were trimmed using Trimmomatic and aligned with Kallisto (v0.43.1) (GRCh38 for human and GRCm38 for mouse). The Bioconductor package Tximport was used to import transcript-level abundance, estimated counts and transcript lengths (v.3.5) (Soneson et al., 2015). Batch correction was performed using limma. Samples were filtered for genes with low counts across samples. The expression level of each gene and the differential expression analysis was performed using the DESeq2 pipeline (v.3.5) (Love et al., 2014). Genes were considered differentially expressed if they had an adjusted p value of ≤ 0.05. Gene Set Enrichment Analysis was performed with the GSEA Jana Desktop tool (Subramanian et al., 2005) and Gene Ontology analysis with Panther (Mi et al., 2019). Heatmaps were generated using Pheatmap. R version 3.4.3 was used.

#### Colony-forming assay

2000 CD34+ sorted cells were plated in MethoCult H4034 Optimum (Stem Cell Technologies) in triplicates and incubated at 37°C, in 5% CO2. Colonies were counted after 14 days.

#### Inhibitor treatment

SEM cells, RS4;11 cells, primary human CD34+ cord blood (Stem Cell Technologies) and primary human CD34+ fetal liver cells were treated with 50nM volasertib-BI6727 (Cambridge BioScience) for 24h, 48h and 72h as indicated and analyzed for viability and cell cycle by flow cytometry. After determination of bezafibrate (Sigma-Aldrich) IC_50_ values on SEM and RS4;11 cells, SEM, RS4;11, PER-494, primary human CD34+ cord blood and primary human CD34+ fetal liver cells were treated with 2 and 4 μM bezafibrate and cell viability determined at 24h and 48h by flow cytometry using Annexin V and Sytox staining.

#### Quantitative RT-PCR

RNA was extracted as described above and cDNA obtained by reverse transcription using iScript (BIO RAD) or PrimeScript cDNA synthesis kit (Takara). Real-time PCR reactions were prepared with TaqMan Fast Master mix in duplicates (assay details are provided in the Key resources table). Samples were analyzed on a Quant Studio RT-qPCR instrument (TaqMan). Clinical data for the human patient samples used for qPCR are provided in Table S4, tab 2. All patients were enrolled in the Interfant-99 trial.

#### Cell cycle analysis

Cycling was assessed with DAPI staining (Sigma-Aldrich) and viability with Zombie dye (Biolegend).

#### Virus preparation and transduction procedure

Lentivirus was produced by co-transfection of Cas9-GFP (Chen et al., 2015) (Addgene: 63592) or sgRNA-BFP (kindly provided by Yusa Kosuke) with MD2-G and psPAX2 (Addgene: 12259 and 12260, respectively) into 293T cells. Viral supernatants were collected after 48h and 72h, filtered through a 0.22 μM membrane and concentrated using LentiX (Clonetech). For gRNA the viral supernatant was used for transductions. The SEM cell line was transduced by spinoculation at 2000rpm for 45min.

#### gRNA selection and verification

gRNAs were designed using the Chop Chop tool (Table S3) and their efficiency assessed using the TIDE assay (Brinkman et al., 2014; Montague et al., 2014; Figure S3F). For that, SEM. Cas9 cells were transduced with sgRNA lentiviral particles and genomic DNA extracted using DNeasy Blood & Tissue Kit (QIAGEN) after 30 – 48h. Genomic DNA fragments were PCR amplified with the following primers and sequenced: ELOVL1 sg1 forw: TCTCCTTTCCAGAGAGGTTCAG; rev: GTGCTTTTTCCACCAAAGGTAG.

#### CRISPR-Cas9 competition assay

SEM wild-type cells were mixed with Cas9-GFP SEM cells in a 1:1 ratio and the mixture transduced with the sgRNA of interest. To ensure efficiency of the assay, a positive and negative sgRNA control was used in every experiment. To assess the impact of the sgRNA, flow cytometric analysis was performed at days 2, 6 and 12. Data were analyzed with FlowJo and FCS Express.

#### siRNA assays

Two ELOVL1-targeted siRNAs (Hama et al., 2021) were used (s34992 and s34993, ThermoFisher Scientific) alongside a negative control (SIC001, Sigma-Aldrich). 1.2 × 10^6^ cells were transfected with 1.5 μM siRNA construct by electroporation and cultured for 2 days before cell viability was quantified by flow cytometry using Annexin V and Sytox staining.

#### Lipidomics

SEM-Cas9 cells were transduced with sgRNAs for ELOVL1 and neg control. 3x10^6^ transduced cells were sorted at day 2 and 4.

Lipids were extracted from cell pellets in 100% isopropanol (MS grade) and extracts further cleared by centrifugation. 10 μL of lipid extract was loaded onto an Accucore C18 column (150 × 2.1mm, Thermo Scientific) fitted with a guard column attached to a Thermo Ultimate 3000 BioRS HPLC. The column was equilibrated in 60% buffer A (50% methanol, 50% water, 10mM ammonium acetate, 0.1% v/v formic acid, 8 μM phosphoric acid) and 40% buffer B (100% isopropanol, 10mM ammonium acetate, 0.1% v/v formic acid, 8 μM phosphoric acid) and the following gradient at 500 μl/min was applied (time/%B): 0.3/40, 3.5/45, 7/75, 25/97. Lipids were eluted into a Q Exactive mass spectrometer (Thermo Scientific) in positive mode with a scan range of 150-2000 in MS1 and data-dependent Top5 MS2 (normalized collision energy 25, isolation window 0.8Da). Other settings were as standard.

Data were processed using Compound Discoverer (Thermo Scientific) with lipid annotations matching an in-house mass list and with MS2 verifications matching an in-house MS2 spectral library.

### Quantification and statistical analysis

Details of the statistical methods used for the RNA-Seq and lipidomics data analyses are provided in the relevant sections of the Methods Details. Details on specific statistical tests, replicate numbers, data display and significance levels are provided in the figure legends. Graphs were prepared in GraphPad Prism (GraphPad software corporation).

## Data Availability

• The RNA-seq data have been deposited at GEO and are publicly available as of the date of publication. The accession number is listed in the Key resources table. • This paper does not report original code. • Any additional information required to reanalyze the data reported in this paper is available from the lead contact upon request.

## Supplemental information

Document S1. Figures S1–S4

Table S1. List of DE genes between human FL HSC/MPPs versus CB HSC/MPPs (tab 1), human FL HSC/MPPs versus LMPPs (tab 2), and murine FL LMPPs versus BM LMPPs (tab 3) and list of DE genes conserved between mouse and human (tab 4), related to Figures 1 and 2

Table S2. Complete list of identified lipids in normal and ELOVL1 knockout SEM cells, related to Figure 4

Table S3. Guide RNAs used for the competition assays, related to Figure 4

Table S4. Clinical data from the samples included from the Andersson et al. (2015) study (tab 1) and from the Agraz-Doblas et al. (2019) study (tab 2), related to Figures 2, 3, S2, and S3

Document S2. Article plus supplemental information
