# Supplementary material for: Defining the fetal origin of MLL-AF4 infant leukemia highlights specific fatty acid requirements
Source: Cell Rep. 2021 Oct 26;37(4):109900. doi: 10.1016/j.celrep.2021.109900 (PMC8567312; doi:10.1016/j.celrep.2021.109900)
Supplement: Document S1. Figures S1–S4 [file mmc1.pdf]

Cell Reports, Volume *37*

Supplemental information

Defining the fetal origin of MLL-AF4 infant leukemia highlights specific fatty acid requirements

Vasiliki Symeonidou, Hélène Jakobczyk, Salem Bashanfer, Camille Malouf, Foteini Fotopoulou, Rishi S. Kotecha, Richard A. Anderson, Andrew J. Finch, and Katrin Ottersbach

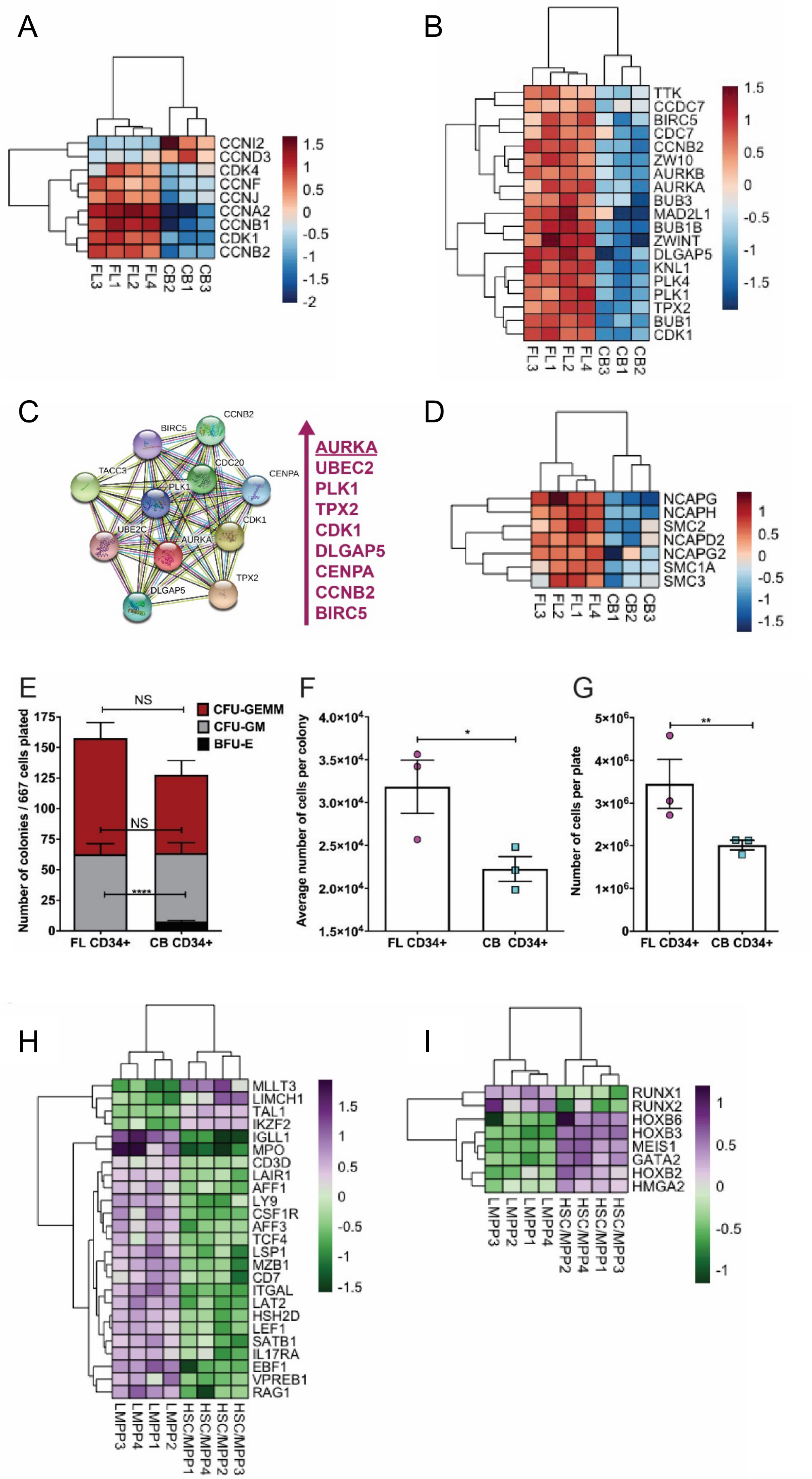

A
CCNI2
CCND3
CDK4
CCNF
CCNJ
CCNA2
CCNB1
CDK1
CCNB2
FL3 FL1 FL2 FL4 CB2 CB1 CB3
1.5 1 0.5 0 -0.5 -1 -1.5 -2
B
TTK
CCDC7
BIRC5
CDC7
CCNB2
ZW10
AURKB
AURKA
BUB3
MAD2L1
BUB1B
ZWINT
DLGAP5
KNL1
PLK4
PLK1
TPX2
BUB1
CDK1
FL3 FL1 FL2 FL4 CB3 CB1 CB2
1.5 1 0.5 0 -0.5 -1 -1.5
C
CCNB2
BIRC5
CDC20
TACC3
CENPA
PLK1
CDK1
UBE2C
AURKA
TPX2
DLGAP5
AURKA
UBEC2
PLK1
TPX2
CDK1
DLGAP5
CENPA
CCNB2
BIRC5
D
NCAPG
NCAPH
SMC2
NCAPD2
NCAPG2
SMC1A
SMC3
FL3 FL2 FL1 FL4 CB1 CB2 CB3
1 0.5 0 -0.5 -1 -1.5
E
NS
CFU-GEMM
CFU-GM
BFU-E
Number of colonies / 667 cells plated
NS
****
FL CD34+
CB CD34+
F
Average number of cells per colony
*
FL CD34+
CB CD34+
G
Number of cells per plate
**
FL CD34+
CB CD34+
H
MLLT3
LIMCH1
TAL1
IKZF2
IGLL1
MPO
CD3D
LAIR1
AFF1
LY9
CSF1R
AFF3
TCF4
LSP1
MZB1
CD7
ITGAL
LAT2
HSH2D
LEF1
SATB1
IL17RA
EBF1
VPREB1
RAG1
LMPP3 LMPP4 LMPP1 LMPP2 HSC/MPP1 HSC/MPP4 HSC/MPP2 HSC/MPP3
1.5 1 0.5 0 -0.5 -1 -1.5
I
RUNX1
RUNX2
HOXB6
HOXB3
MEIS1
GATA2
HOXB2
HMGA2
LMPP3 LMPP2 LMPP1 LMPP4 HSC/MPP2 HSC/MPP4 HSC/MPP1 HSC/MPP3
1 0.5 0 -0.5 -1

Figure S1: Cell proliferation is a predominant feature of the fetal liver-derived cells. Related to Figure 1.

(A) Heatmap of cyclins (CCN) and cyclin-dependent kinases (CDK). **(B)** Heatmap of other genes critical for cell cycle/division. **(C)** AURKA and its protein interactome with a list of genes that were more highly expressed in the fetal liver-derived HSC/MPPs (Graph obtained from string-db.org). **(D)** Heatmap of cohesins and condensins. **(E)** Colonies produced by CD34+ human fetal liver (FL) and cord blood (CB) cells after two weeks in methylcellulose under myeloid conditions. Number of cells per colony **(F)** and number of cells per plate **(G)** produced by CD34+ human fetal liver (FL) and cord blood (CB) cells after two weeks in methylcellulose under myeloid conditions. n=3 with triplicates per biological replicate. Data are shown as mean ±SD, Anova test was performed. NS-not significant; *p<0.05; **p<0.01; ****p<0.0001. **(H)** Heatmap of genes required for lymphoid and myeloid commitment, differentially expressed between fetal liver-derived LMPPs compared to fetal liver-derived HSC/MPPs. **I** Heatmap of genes associated with a stem cell signature, differentially expressed between fetal liver-derived LMPPs compared to fetal liver-derived HSC/MPPs.

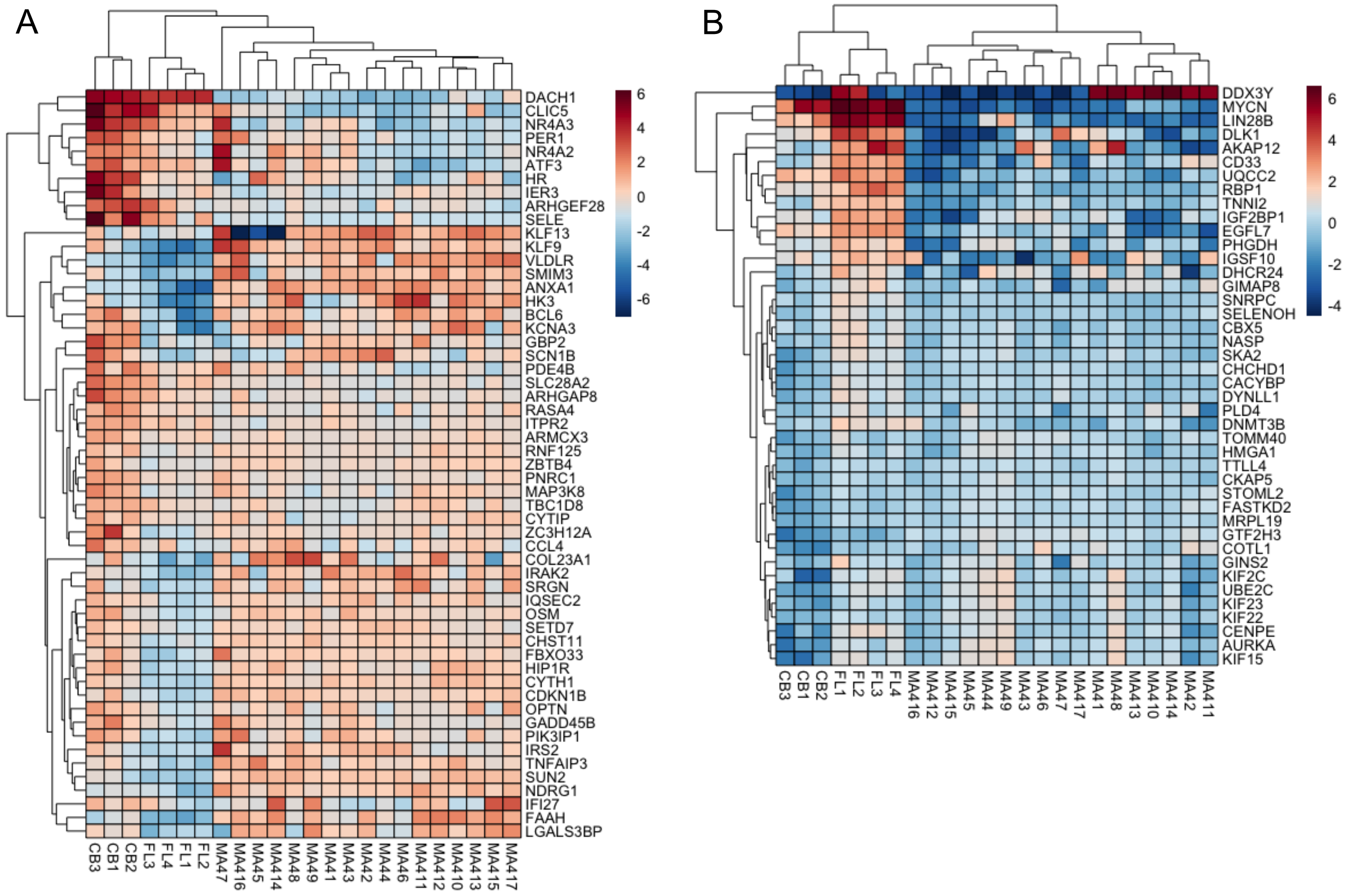

Figure S2: Heatmap of common neonatal/adult genes and common fetal genes that are not maintained in infant blasts. Related to Figure 2.

(A) Heatmap of the genes commonly overrepresented between CB (human) and BM (murine) derived cells (human and murine) showing their expression pattern in human CB, FL and infant MLL-AF4+ blasts. **(B)** Heatmap of the genes commonly overrepresented between FL-derived cells (human and murine) that are downregulated in the blasts. It should be noted that genes HMGA1 and AURKA are expressed at similar levels between FL and blasts but were not investigated in this study.

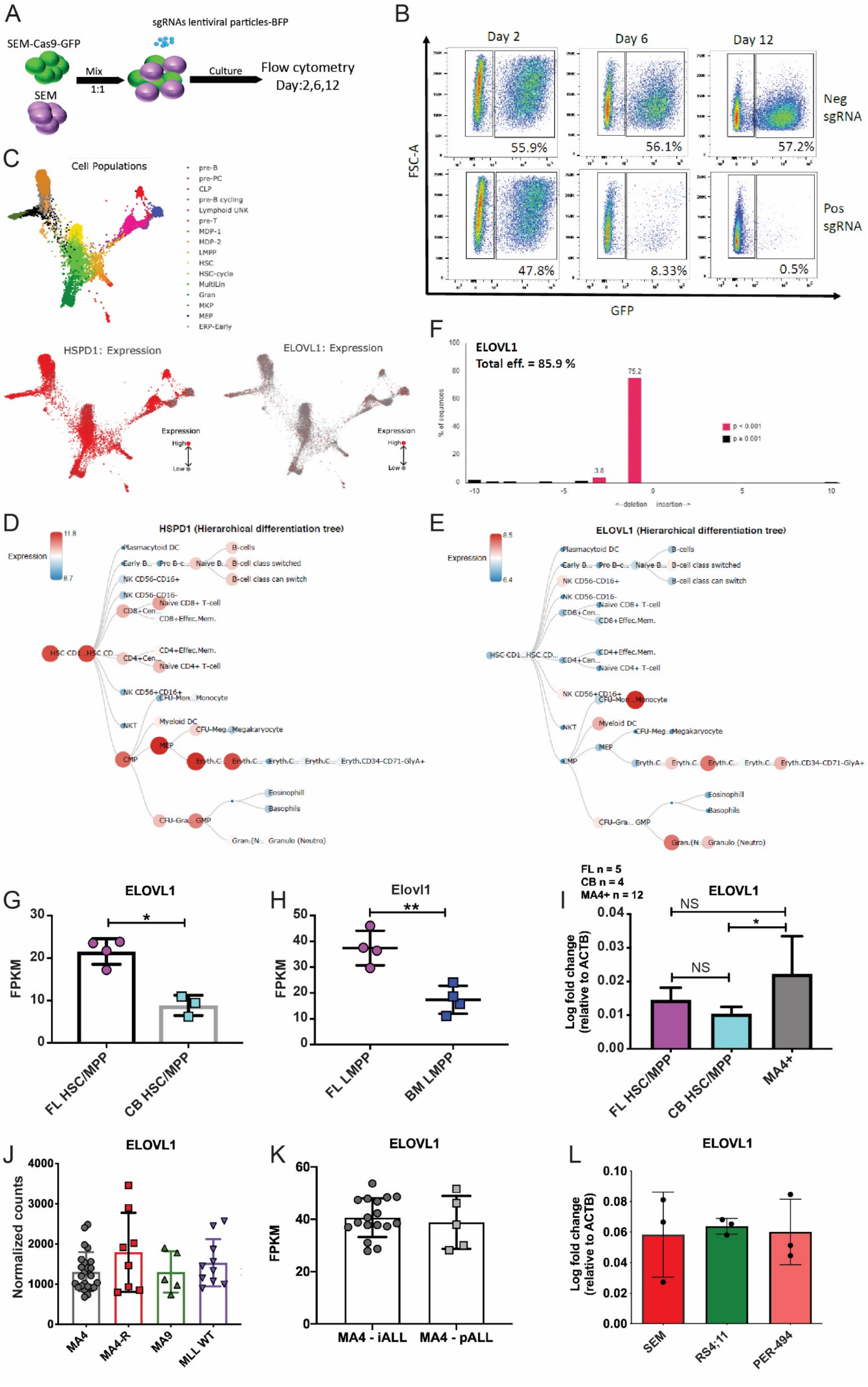

A
SEM-Cas9-GFP
SEM
Mix
1:1
sgRNAs lentiviral particles-BFP
Culture
Flow cytometry
Day:2,6,12
B
Day 2
Day 6
Day 12
Neg sgRNA
Pos sgRNA
55.9%
56.1%
57.2%
47.8%
8.33%
0.5%
FSC-A
GFP
C
Cell Populations
pre-B
pre-PC
CLP
pre-B cycling
Lymphoid UNK
pre-T
MDP-1
MDP-2
LMPP
HSC
HSC-cycle
MultiLin
Gran
MKP
MEP
ERP-Early
HSPD1: Expression
ELOVL1: Expression
Expression
High
Low
F
ELOVL1
Total eff. = 85.9 %
75.2
3.8
% of sequences
p < 0.001
p ≥ 0.001
<--deletion insertion-->
D
HSPD1 (Hierarchical differentiation tree)
E
ELOVL1 (Hierarchical differentiation tree)
Expression
G
ELOVL1
FPKM
FL HSC/MPP
CB HSC/MPP
H
Elovl1
FPKM
FL LMPP
BM LMPP
I
FL n = 5
CB n = 4
MA4+ n = 12
ELOVL1
NS
Log fold change (relative to ACTB)
FL HSC/MPP
CB HSC/MPP
MA4+
J
ELOVL1
Normalized counts
MA4
MA4-R
MA9
MLL WT
K
ELOVL1
FPKM
MA4 - iALL
MA4 - pALL
L
ELOVL1
Log fold change (relative to ACTB)
SEM
RS4;11
PER-494

Figure S3: Identification of disease-relevant genes and verification of *ELOVL1* expression. Related to Figure 4.

(A) Experimental outline for competition assay. **(B)** flow cytometry profile of the positive and negative control used in the competition assay. **(C)** Human hematopoietic bone marrow cell population assignment of scRNA-Seq data within the Human Cell Atlas with *HSPD1* and *ELOVL1* expression overlaid. Data obtained from the Human Cell Atlas Bone Marrow Single-Cell interactive Web portal (http://www.altanalyze.org/ICGS/HCA/splash.php). Red color indicates higher expression. **(D)** *HSPD1* and **(E)** *ELOVL1* expression in BloodSpot (https://servers.binf.ku.dk/bloodspot/). **(F)** Validation of ELOVL1 sgRNA using TIDE assay, showing the proportion of deletions and insertions detected by genomic DNA sequencing. **(G)** *ELOVL1* expression in human fetal liver (FL) and cord blood (CB) derived HSC/MPPs and **(H)** *Elovl1* expression in murine fetal liver (FL) and adult bone marrow (BM) derived LMPPs. RNA sequencing data are shown as mean ±SD, each dot represents a sample. FPKM, Fragments Per Kilobase of transcript per Million. **(I)** qPCR validation of expression of *ELOVL1* in human fetal liver (FL) and cord blood (CB) derived HSC/MPPs and infant blasts (MA4+). FL: n=5; CB: n=4; MA4+: n=12. Data are shown as mean +SD, Anova test was performed. NS-not significant; *p<0.05; **p<0.01. *ELOVL1* expression in the RNA-sequencing datasets by **(J)** Agraz-Doblas *et al.*, 2019 and **(K)** Andersson *et al.*, 2015. MA4=MLL-AF4; MA4-R=relapsed MA9=MLL-AF9; MLL WT=MLL germline B-ALL; iALL=infant B-ALL; pALL=pediatric B-ALL. **(L)** *ELOVL1* expression in three human MLL-AF4+ B-ALL-derived cell lines.

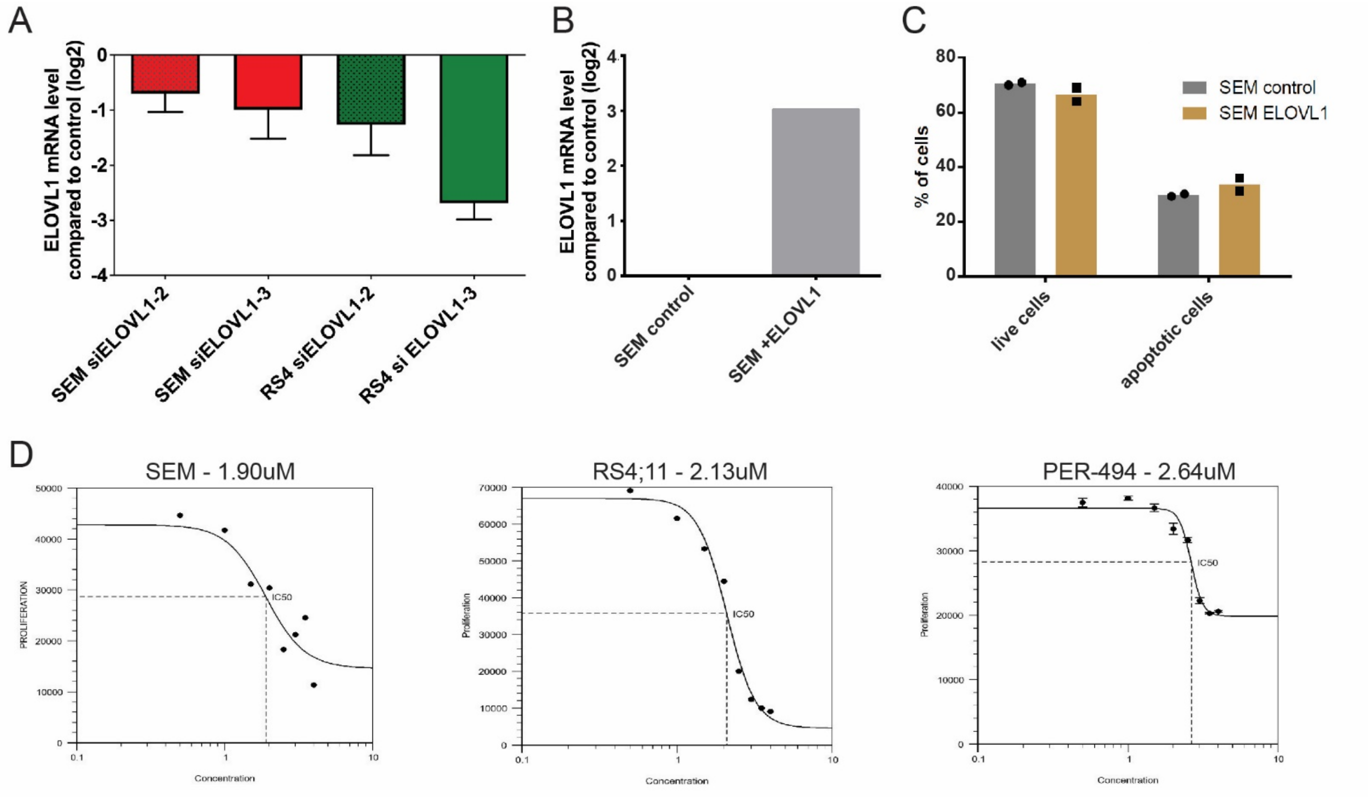

Figure S4: ELOVL1 and very long chain fatty acid synthesis inhibition. Related to Figure 4.

(A) Knockdown levels of *ELOVL1* in SEM and RS4;11 cells. Expression is shown relative to siControl. **(B)** Overexpression levels of *ELOVL1* in SEM cells. Expression is shown relative to control. **(C)** Viability of *ELOVL1*-overexpressing SEM cells after 48h in culture. 'Live' cells are defined as AnnexinV- Sytox-, and 'apoptotic' cells as AnnexinV+ Sytox+/-. **(D)** IC50 curves of bezafibrate treatment in three human MLL-AF4+ B-ALL cell lines. The IC50 value for each cell line is stated above each curve.
